# Characterization of high-H_2_O_2_-tolerant bacterial cytochrome P450 CYP105D18: insights into papaverine N-oxidation

**DOI:** 10.1107/S2052252521005522

**Published:** 2021-06-29

**Authors:** Bashu Dev Pardhe, Hackwon Do, Chang-Sook Jeong, Ki-Hwa Kim, Jun Hyuck Lee, Tae-Jin Oh

**Affiliations:** a Department of Life Science and Biochemical Engineering, Graduate School, SunMoon University, Asan 31460, Republic of Korea; b Research Unit of Cryogenic Novel Material, Korea Polar Research Institute, 26, Songdomirae-ro, Yeonsu-gu, Incheon 21990, Republic of Korea; c Department of Polar Sciences, University of Science and Technology, Incheon 21990, Republic of Korea; d Genome-based BioIT Convergence Institute, Asan 31460, Republic of Korea; e Department of Pharmaceutical Engineering and Biotechnology, SunMoon University, Asan 31460, Republic of Korea

**Keywords:** CYP105D18, papaverine *N*-oxide, H_2_O_2_ tolerance, *Streptomyces laurentii*, enzyme mechanisms, crystal morphology, co-crystals

## Abstract

The bacterial CYP105 family is involved in secondary metabolite biosynthetic pathways and plays essential roles in the biotransformation of xenobiotics. This study investigates the newly identified H_2_O_2_-mediated CYP105D18 from *Streptomyces laurentii* as the first bacterial CYP for N-oxidation. The catalytic efficiency of CYP105D18 for papaverine N-oxidation was 1.43 s^−1^ µ*M* ^−1^. The heme oxidation rate (*k*) was low (<0.3 min^−1^) in the presence of 200 m*M* H_2_O_2_. This high H_2_O_2_ tolerance capacity of CYP105D18 led to higher turnover prior to heme oxidation. Additionally, the high-resolution papaverine complexed structure and substrate-free structure of CYP105D18 were determined. Structural analysis and activity assay results revealed that CYP105D18 had a strong substrate preference for papaverine because of its bendable structure. These findings establish a basis for biotechnological applications of CYP105D18 in the pharmaceutical and medicinal industries.

## Abstract 2

The crystal structure of CYP105D18 and its unique structural features for papaverine N-oxidation are presented.

## 1. Introduction   

Cytochrome P450 (CYP) enzymes comprise a large superfamily of heme *b*-containing enzymes expressed in a variety of species. These enzymes catalyze diverse reactions, including hy­droxy­lation, alcohol and carbonyl oxidation, epoxidation, de­methyl­ation, N-oxidation, ring cleavage, coupling, and formation of a vast number of endogenous and exogenous compounds, such as steroids, fatty acids, xenobiotics and other drugs (Isin & Guengerich, 2007 ▸). CYPs are involved in regio- and stereo-specific reactions with substrate flexibility, making them suitable targets for biotechnological applications (Cia­ramella *et al.*, 2020 ▸). The complex mechanism of CYP reactions involves specific redox partners and costly cofactors, thereby limiting the broad utility of CYPs in nature. However, some CYP monooxygenases and per­oxy­genases support the shunt pathway using an oxygen surrogate system such as hydrogen peroxide (H_2_O_2_) and organic surrogates such as cumene hydro­peroxide or iodo­syl­benzene for efficient catalysis of different substrates (Wei *et al.*, 2019 ▸; Munro *et al.*, 2018 ▸). Implementation of this system may be economically viable, with some P450s evolving to use H_2_O_2,_ but is typically inefficient and may lead to heme oxidation and enzyme destabilization or inactivation (Ciaramella *et al.*, 2020 ▸; Munro *et al.*, 2018 ▸). In addition, the shape and size of the substrate-binding pocket may hinder the formation of highly reactive compounds using the shunt pathway for efficient catalysis (Ciaramella *et al.*, 2020 ▸; Wei *et al.*, 2019 ▸).

The general ability of CYPs to utilize H_2_O_2_ has been demonstrated for many human CYPs and bacterial CYPs. Human CYP1A2 supports H_2_O_2_ shunting for efficient oxidation of aromatic amines (Anari *et al.*, 1997 ▸), and CYP3A4 and CYP2D6 can convert pinacidil (a vasodilator drug) into its corresponding amine using H_2_O_2_ (Zhang *et al.*, 2002 ▸). The major human drug-metabolizing CYPs, *i.e.* CYP2C9, CYP2C19, CYP2D6 and CYP1A2, can also support different oxygen surrogate systems, and their catalysis function is based on the various substrates used (Strohmaier *et al.*, 2020 ▸). Typically, members of the bacterial CYP152 family, including P450BSβ, P450SPα and OleTJe, have been shown to utilize H_2_O_2_ for efficient fatty acid hy­droxy­lation (Munro *et al.*, 2018 ▸).

Papaverine, an iso­quinoline alkaloid present in opium, causes smooth muscle relaxation and has been used to treat vasospasm. Moreover, papaverine has been administered as an alternative to nonsteroidal anti-inflammatory drugs in some patients to treat renal colic pain, migraine headaches, schizophrenia, cancers and even viral infections (Shen *et al.*, 2019 ▸; Aggarwal *et al.*, 2020 ▸). Despite the medicinal importance of papaverine, this compound has many adverse effects. However, modified papaverine may have better efficacy and reduced cytotoxicity and can easily be metabolized by the body. Chemical modification of papaverine for C—H functionalization, N-oxidation (Egbewande *et al.*, 2019 ▸) and O-de­methyl­ation has been reported (Brossi & Teitel, 1970 ▸). These classical approaches are time-consuming and costly and may suffer from poor regio-selectivity compared with enzyme-catalyzed reactions. Shen *et al.* (2019 ▸) reported an efficient biocatalyst, CYP105D1, for the 6-*O*-de­methyl­ation of papaverine.

The CYP105 family is involved in biosynthetic pathways of secondary metabolites and biotransformation of xenobiotic compounds. For example, CYP105D7 from *Streptomyces avermitilis* catalyzes the hydration of 1-de­oxy­pentalenic acid at the C1 position, isoflavone and daidzein at the 3 position and diclofenac at the C4′ position (Takamatsu *et al.*, 2011 ▸; Pandey *et al.*, 2010 ▸; Xu *et al.*, 2015 ▸). Additionally, CYP105A1 from *Streptomyces griseous* converts vitamin D_3_ (VD_3_) to 1,25-di­hydroxy vitamin D3, a hormonally active form, via two sequential hy­droxy­lations (O’Keefe *et al.*, 1988 ▸; Hayashi *et al.*, 2008 ▸). CYP105P1 and CYP105D6 are also involved in the biosynthesis of filipin (Xu *et al.*, 2010 ▸). Although the CYP105 family has diverse functions, all members share a common CYP structure within a relative mean squared deviation of 2 Å, indicating that further close structural investigation is required to determine the regio- and/or stereo-specific substrate recognition and chemistry (Lee *et al.*, 2016 ▸).

In this study, we aimed to determine biochemical and structural information for proteins in the CYP105 family. We studied CYP, annotated as CYP105D18, from *Streptomyces laurentii*. We also determined the substrate-free and papaverine-complexed crystal structures of CYP105D18 at resolutions of 1.7 and 2.0 Å, respectively. To the best of our knowledge, this is the first report of an N-oxidation reaction using bacterial CYP.

## 2. Results and discussion   

### 2.1. Functional characterization of CYP105D18   

**Figure 1:**
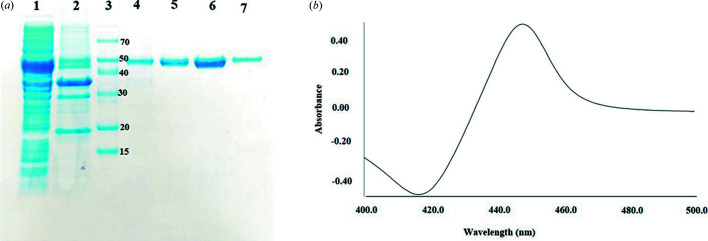
(*a*) Sodium do­decyl sulfate-polyacryl­amide gel electrophoresis analysis of the purification of CYP105D18 [1, crude extract; 2, insoluble fraction; 3, marker (kDa); 4, 10 m*M* imidazole fraction; 5, 100 m*M* first fraction; 6, 100 m*M* second fraction and 7, 500 m*M* imidazole fraction]. The 100 m*M* imidazole second fraction (sample 6) was concentrated for *in vitro* reaction. The molecular weight of CYP105D18 is 44.3 kDa, and the protein was overexpressed using the pET28 vector in the C41 host. (*b*) Di­thio­nite reduced the CO-bound form of CYP105D18.

**Figure 2:**
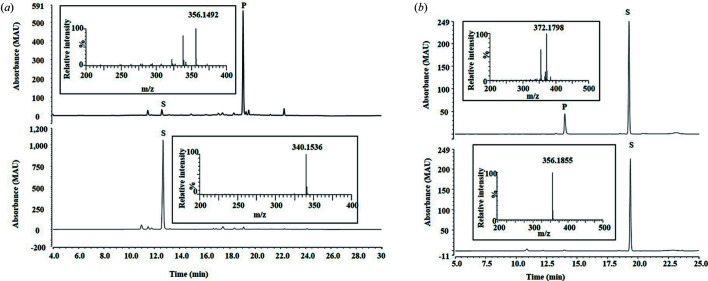
HPLC chromatogram and LC-MS spectra (inside the box) for (*a*) papaverine and (*b*) tetra­hydro­palmatine. The reaction (above) was performed with 400 µ*M* substrate and 3 µ*M* CYP105D18 in the presence of 40 m*M* H_2_O_2_ for 1 h. A similar reaction condition was used for the control (below), with the use of 3 µ*M* heat-deactivated CYP105D18 (positive control) and without the CYP105D18 (negative control). The positive and negative controls showed a similar HPLC chromatogram without any product formation.

Phylogenetic tree analysis of CYP105D18 revealed that it had the closest relationships with functionally characterized CYP105D7 (71.1%) and CYP105D1 (64.9%; Fig. S1 of the supporting information). CYP105D1 was well characterized for biotransformation of iso­quinoline alkaloids (Shen *et al.*, 2019 ▸). So, we speculated that this protein is involved in the biotransformation of different iso­quinoline alkaloids (*e.g.* papaverine, THP, berberine and palmatine). CYP105D18 was cloned, overexpressed and purified. The theoretical molecular weight was 44.3 kDa, and a single band at ∼50 kDa (with a His-tag from pET28) was obtained by sodium do­decyl sulfate-polyacryl­amide gel electrophoresis [Fig. 1 ▸(*a*)]. Purified CYP105D18 showed a Soret peak at 418 nm in an oxidized form. The carbon-monoxide-bound and di­thio­nite-reduced form of CYP105D18 exhibited maximum absorption at a wavelength of 449 nm, one of the important spectral characteristics of CYP450 [Fig. 1 ▸(*b*)]. The purity of a CYP can be evaluated from its *R_z_* value, calculated as the ratio of absorbance (*A*) at λ_max_ of the Soret band to the *A* value at 280 nm. The purified enzyme had an *R_z_* value of 1.45, indicating high purity (Lee *et al.*, 2016 ▸). The redox partner proteins Pdx and PdR were expressed and purified to single bands corresponding to 11.4 and 45.6 kDa, respectively (Fig. S2). The four iso­quinoline alkaloids with different structures (listed in Fig. S3) were screened for *in vitro* biotransformation by CYP105D18. There was no *in vitro* biotransformation by CYP105D18 for papaverine, palmatine, THP or berberine using the redox partners Pdx/PdR from the P450cam system and/or spinach Fdx/FdR (data not shown). CYP105D18 supported the N-oxidation of papaverine and hy­droxy­lation of tetra­hydro­palmatine using the oxygen surrogate H_2_O_2_. High-performance liquid chromatography (HPLC) analysis of papaverine revealed product formation at a retention time of 18.2 min, equivalent to hy­droxy­lation/N-oxidation in liquid chromatography-mass spectrometry (LC-MS) analysis [Fig. 2 ▸(*a*)]. The HPLC results showed an additional peak at a retention time of 22.2 min, which could not be identified because of the poor degree of ionization by LC-electrospray ionization-MS. The major product was purified and characterized by nuclear magnetic resonance analysis as papaverine *N*-oxide (Fig. S4). Similarly, the tetra­hydro­palmatine product catalyzed by CYP105D18 in the presence of H_2_O_2_ showed a single peak at a retention time of 13.5 min in the HPLC analysis [Fig. 2 ▸(*b*)]. The LC-MS data revealed the mono-hy­droxy­lation (mass, 372.179 g mol^−1^) of tetra­hydro­palmatine. The maximum product conversion for tetra­hydro­palmatine under optimum conditions was less than 12%; therefore, we did not purify the product for structural analysis. The results showed no support from H_2_O_2_ as an oxygen surrogate partner for biotransformation of berberine and palmatine by CYP105D18.

To support the *in vitro* biotransformation of papaverine by CYP105D18, we performed the substrate-binding experiment. Among the four substrates, only papaverine induced the spectral shift, which was at 422 nm (Fig. S5). Even though THP was catalyzed by CYP105D18 using H_2_O_2_ as co-substrate, there was no spin-shift observed. The binding of 4-(pyridine-3-yl) benzoic acid and 4-(pyridine-2-yl) benzoic acid to CYP199A4 exhibits type II UV–vis spectral changes showing a greater shift in the Soret wavelength (422 versus 424 nm) (Podgorski *et al.*, 2020 ▸). Papaverine was also modified in the pyridine ring (N-oxidation) by CYP105D18 and exhibits a similar pattern of spectral shift at 422 nm. In addition, there was a spectral difference in the δ band between substrate-bound and unbound forms. Papaverine-bound spectra showed a Soret band at 322 nm, which was not observed in the binding of 4-(pyridine-3-yl) benzoic acid and 4-(pyridine-2-yl) benzoic acid to CYP199A4 (Podgorski *et al.*, 2020 ▸).

This is the first report of N-oxidation of a compound by bacterial CYP. However, human CYP has been reported to catalyze the N-oxidation of drugs during drug modification by liver microsomes. CYP2E1 is the main microsomal enzyme involved in the N-oxidation of nicotinamide (Real *et al.*, 2013 ▸), and CYP2C9, CYP2C19 and CYP3A4 exhibit different affinities for N-oxidation of the antifungal drug voriconazole during its metabolism in liver microsomes (Hyland *et al.*, 2003 ▸). In addition, the fungal aromatic per­oxy­genase (AaP) was reported to catalyze the N-oxygenation of pyridine and related compounds (Ullrich *et al.*, 2008 ▸). Nitro­gen-rich heterocyclic compounds are used to treat various human diseases, and these motifs are found in many iso­quinoline alkaloid drugs (Egbewande *et al.*, 2019 ▸). The *N*-oxide derivatives of iso­quinoline alkaloids are structurally and functionally related to the original compound and are reported to have good pharmacological properties (Dembitsky *et al.*, 2015 ▸). Bremner & Wiriyachitra (1973 ▸) reported the chemical synthesis of papaverine *N*-oxide from papaverine using chloro­form and *m*-chloro­perbenzoic acid. The photochemical synthesis of papaverine *N*-oxide was reported by Girreser *et al.* (2003 ▸). However, chemical and photochemical methods are time-consuming and exhibit poor yields, as reported previously. For example, papaverine was treated with aqueous H_2_O_2_ at 70°C for up to 10 h to produce papaverine *N*-oxide (Girreser *et al.*, 2003 ▸). In contrast, we showed that CYP105D18, in the presence of a stoichiometric amount of H_2_O_2_, efficiently transformed papaverine to papaverine *N*-oxide, providing important insight into chemical methods.

### 2.2. H_2_O_2_ stability for CYP105D18 and optimization for papaverine N-oxidation   

**Figure 3:**
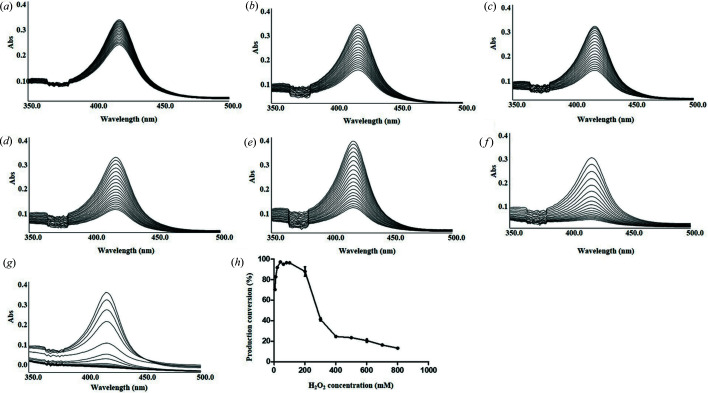
Heme oxidation pattern of CYP105D18 in the presence of different concentrations of H_2_O_2_. (*a*) 1 m*M*, (*b*) 5 m*M*, (*c*) 10 m*M*, (*d*) 20 m*M*, (*e*) 40 m*M*, (*f*) 100 m*M* and (*g*) 200 m*M* H_2_O_2_. The concentration of CYP was fixed at 3 µ*M*. The decrease in the Soret peak (417 nm) was measured at 90 s intervals for 30 min. Optimization of H_2_O_2_ concentrations for *in vitro* papaverine N-oxidation by CYP105D18 (*h*). The reaction conditions were as follows: 3 µ*M* CYP, 400 µ*M* substrate and 5–800 m*M* H_2_O_2_. The means and standard deviations were obtained from three independent reactions with similar conditions.

Only H_2_O_2_ supported CYP105D18-dependent N-oxidation of papaverine; therefore, we analyzed the oxidative degradation of heme under different concentrations of H_2_O_2_ (0.1–200 m*M*). Unexpectedly, the rate constant ‘*k*’ was low, even in the presence of higher concentrations of H_2_O_2_, suggesting the high tolerance capacity of CYP105D18 for H_2_O_2_ (Table 1 ▸). The decrease in Soret absorbance at 417 nm 90 s after treatment with 0.1–200 m*M* H_2_O_2_ for 30 min is shown in Figs. 3 ▸(*a*)–3(*g*). The well established P450 per­oxy­genase P450s SPα (CYP152B1 from *Sphingomonas paucimobilis*), BSβ (CYP152A1 from *Bacillus subtilis*) and OleT (CYP152L1 from *Jeotgalicoccus sp*.) have been reported to replace the acid–alcohol amino acid pair, which is used for proton relay to iron–oxo species to facilitate the catalytic cycle (Munro *et al.*, 2018 ▸). General CYPs with conserved acid–alcohol pairs (*i.e.* BM3, CYP15B1 and CYP121A1) have also been reported to utilize H_2_O_2_. The steroid hy­droxy­lase CYP154C8 surprisingly functioned in the presence of a high concentration of H_2_O_2_, as reported in our previous work (Dangi *et al.*, 2018 ▸). The stability of heme also plays a crucial role in the efficient catalysis of the CYP. For example, P450116B5hd showed higher tolerance than P450BMP (Ciaramella *et al.*, 2020 ▸). Here, we report the first CYP105 subfamily enzyme showing tolerance for H_2_O_2_. Indeed, CYP105D18 was found to utilize H_2_O_2_ for efficient N-oxidation of papaverine with high stabil­ity. The heme oxidation rate constant *k* was lower than those of the steroid hy­droxy­lases CYP154C8, CYP152L1 and P450116B5hd. The heme dissociation rate constant was less than 0.3 min^−1^, even in the presence of 200 m*M* H_2_O_2_. Enzymes with high stability in the presence of H_2_O_2_ may exhibit more frequent turnover before definitive heme oxidation.

The heme oxidation rate *k* was low, even in the presence of high concentrations of H_2_O_2_. The conversion of papaverine to papaverine *N*-oxide by 3 µ*M* CYP105D18 with different H_2_O_2_ concentrations is shown in Fig. 3 ▸(*h*). A product conversion rate of 70% was observed with 5 m*M* H_2_O_2_. The product conversion was more than 95% between 40 and 200 m*M* H_2_O_2_. The heme dissociation constant was less than 0.014 min^−1^, and product conversion was greater than 97% using 40 m*M* H_2_O_2_ within 30 min. Therefore, 40 m*M* H_2_O_2_ was used as the optimum concentration to evaluate the enzyme kinetics. The product conversion decreased sharply when the the H_2_O_2_ concentration was greater than 200 m*M*, and more than 10% conversion was observed in the presence of 800 m*M* H_2_O_2_.

**Figure 4:**
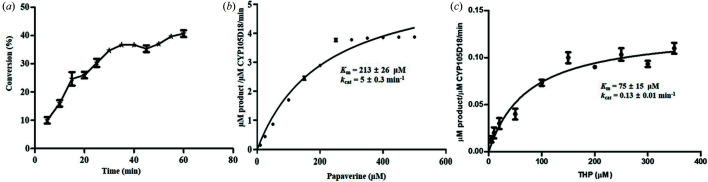
(*a*) Time-dependent conversion of papaverine to papaverine *N*-oxide. Conversion was measured every 5 min for 1 h using 1 µ*M* CYP. The reactions were initiated by 40 m*M* H_2_O_2_. (*b*) Hyperbolic fit for papaverine *N*-oxide. (*c*) Hyperbolic fit for tetra­hydro­palmatine (THP). Average *K*_m_ and *k*_cat_ values were calculated with standard deviations from three independent experiments performed under similar conditions.

Time-dependent *in vitro* conversion of papaverine *N*-oxide by CYP105D18 is shown in Fig. 4 ▸(*a*). The *K*_m_ and *k*_cat_ values for papaverine were estimated to be 213 ± 26 µ*M* and 5 ± 0.3 min^−1^, respectively [Fig. 4 ▸(*b*)]. Similarly, *K*_m_ and *k*_cat_ values for tetra­hydro­palmatine were 75 ± 15 µ*M* and 0.13 ± 0.01 min^−1^, respectively [Fig. 4 ▸(*c*)]. The catalytic efficiency (*k*_cat_/*K*_m_) for papaverine was much higher (1.43 s^−1^ µ*M* ^−1^) in comparison with tetra­hydro­palmatine (0.11 s ^−1^ µ*M* ^−1^).

### 2.3. Overall substrate-free structure of CYP105D18   

**Figure 5:**
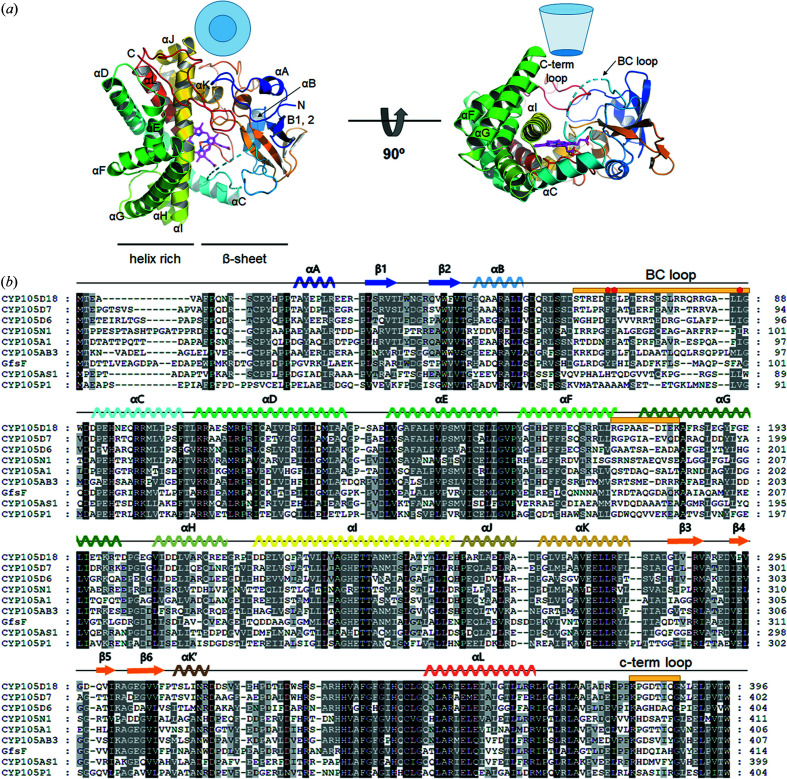
(*a*) Ribbon diagram of the CYP105D18 structure. Rainbow coloring from blue to red indicates the N- to C-terminal positions of residues in the model. The right panel is rotated by 90° relative to the left panel, such that the side of the substrate-binding funnel in the right panel orients toward the reader in the left panel. The nomenclature of the secondary helices and β-sheets was chosen from previous studies. (*b*) Multiple sequence alignment with other homologous CYPs. CYP105D18 from *S. laurentii*, CYP105D7 and CYP105D6/PteD from *Streptomyces sp.*, CYP105D6 from *S. avermitilis*, CYP105N1 from *S. coelicolor*, CYP105A1 from *S. griseolus*, CYP105AB3 from *Nonomuraea recticatena*, GfsF from *S. gramino­faciens*, CYP105AS1 from *Amycolatopsis orientalis*, and CYP105P1 from *S. avermitilis*. Secondary structural elements are shown above the aligned sequences and colored according to the scheme in Fig. 5 ▸(*a*). Regions for the active site are marked with orange sticks, and conserved residues at the B/C loop and turn of αF–αG are marked by red circles.

Structural investigation of CYP105D18 was conducted to obtain detailed structural information related to the active site. Crystallization experiments for full-length CYP105D18 yielded crystals in the space group *C*2 diffracting to 1.7 Å. A final model was obtained with *R* factor and *R*_free_ values of 0.16 and 0.21, respectively, and a Molprobity score of 1.48 (Williams *et al.*, 2018 ▸). A Ramachandran plot analysis of the refined model showed that 96.55 and 0% of non-glycine residues were in favored regions and outliers, respectively. The final model of CYP105D18 exhibited electron density for most of the molecule, except for the N-terminus (amino acids 1–3) and a loop containing residues 73–85, which connected αB and αC [dotted line in Fig. 5 ▸(*a*)]. The asymmetric unit had one molecule of CYP105D18. Consistent with this observation, size exclusion chromatography analysis of CYP105D18 revealed that the protein existed as a monomer in solution (Fig. S6). The coordinates were deposited in the Protein Data Bank under entry 7di3 (substrate-free structure). Data collection and refinement statistics are summarized in Table S4 of the supporting information.

As observed in the structures of the CYP105 family, CYP105D18 can be divided into two units: β-sheet-containing units and helix-rich units composed of helices αC–αJ [Fig. 5 ▸(*a*)]. A long αI was located in the middle between the two units and connected two units. CYP105D18 contained a heme surrounded by loops from the β-sheet-containing unit and αC, αI and αL from the helix-rich unit. The last C-terminus long loop starting from Lue366 covered the concave active site. As shown in the phylogenetic tree, CYP105D18 had the highest homology with the CYP105D subfamily, including CYP105D4, CYP105D7, CYP105D8 and CYP105D1. Consistent with this, structural similarities generated from the *DALI* server (http://ekhidna2.biocenter.helsinki.fi/dali/) showed high similarity with structures of CYP105D7 and CYP105D6 (PetD) from *S. avermitilis* (*Z*-scores: 53.7 and 53.0, respectively; Table 2 ▸) (Xu *et al.*, 2015 ▸, 2010 ▸).

CYP105D18 forms a deep, concave and funnel-like active site located in the center between the β-sheet and helix-rich units; at the bottom of the active site, the heme plane is located vertically with respect to the active site funnel (Fig. 5 ▸). The active site funnel shape is composed of a B/C loop (residues 173–187), turn region between αF and αG, and C-terminal loop (residues 336–396). Although the CYP105 family shares high structural homology but has diverse substrates, residues from these three regions are barely conserved [Fig. 5 ▸(*b*)], indicating that these regions are essential for substrate selectivity for the CYP family. A previous study of CYP105D7 indicated that the arginine residues (Arg70, Arg88 and Arg190) in the B/C loop and αG of the substrate-binding pocket are conserved in the CYP105 family and may be important residues for substrate recognition (Xu *et al.*, 2015 ▸). Unlike CYP105D7, CYP105D18 contains Ser63 and Phe184, corresponding to Arg70 and Arg190 of CYP105D7. This suggests that the residual recognition of the substrate at the active site of CYPs is more complicated and varied.

### 2.4. Structural feature for the H_2_O_2_ stability of CYP105D18   

**Figure 6:**
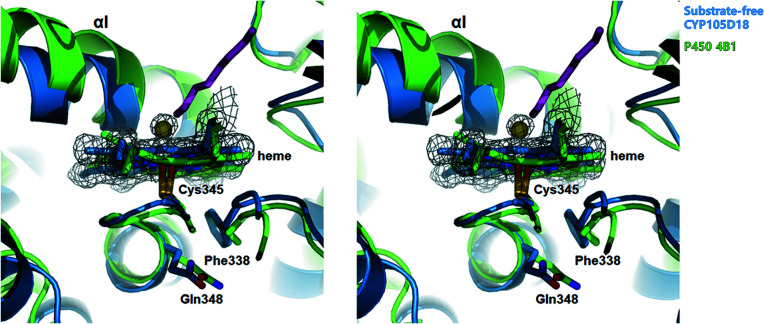
Superposition of CYP105D18 with H_2_O_2_-insensitive rabbit P450 4B1. Residues corresponding to Cys448, Phe441 and Gln451 of P450 4B1 are shown using sticks. The 2*F*_o_ − *F*_c_ electron density map for the heme and water molecule for CYP105D18 is shown as a gray mesh (contoured at the 2.0σ level). The octane molecule from P450 4B1 is presented as a purple stick.

Previous studies have reported that human P450 1A2 is thiol-insensitive to H_2_O_2_. The molecular dynamics simulation using rabbit P450 4B1 as a model suggested that the Gln451 of P450 4B1 is an essential residue for the thiol sensitivity of P450 (Albertolle *et al.*, 2019 ▸). Gln451 in P450 4B1 showed heme thiol­ate and sulfenic acid-dependent open and closed conformations, respectively. These conformational changes alter the solvent accessibility of heme-thiol­ate Cys488. Once the Cys488 was oxidized to sulfenic acid, the Gln451 forms an NH–π bond with Phe441 for closed conformation, limiting the accessibility of oxidizing agents to the protein (Albertolle *et al.*, 2019 ▸). Consistent with this, H_2_O_2_-stable CYP105D18 shows high sequential and structural similarity with P450 4B1. Across from the heme molecule are Phe338 and Gln348, which correspond to Phe441 and Gln451 of P450 4B1 and show a closed conformation. Notably, the substrate-free CYP105D18 shows a small blob on the heme molecules, as depicted in Fig. 6 ▸, indicating that the heme of CYP105D18 is oxidized. A similar mechanism with the P450 4B1 access region to the heme region of CYP105D18 may involve conformational changes upon oxidation, and this closed conformation probably results in the H_2_O_2_ resistance of CYP105D18 (Fig. 6 ▸).

### 2.5. Comparison of substrate-binding pockets between substrate-free and papaverine complexes with CYP105D18   

**Figure 7:**
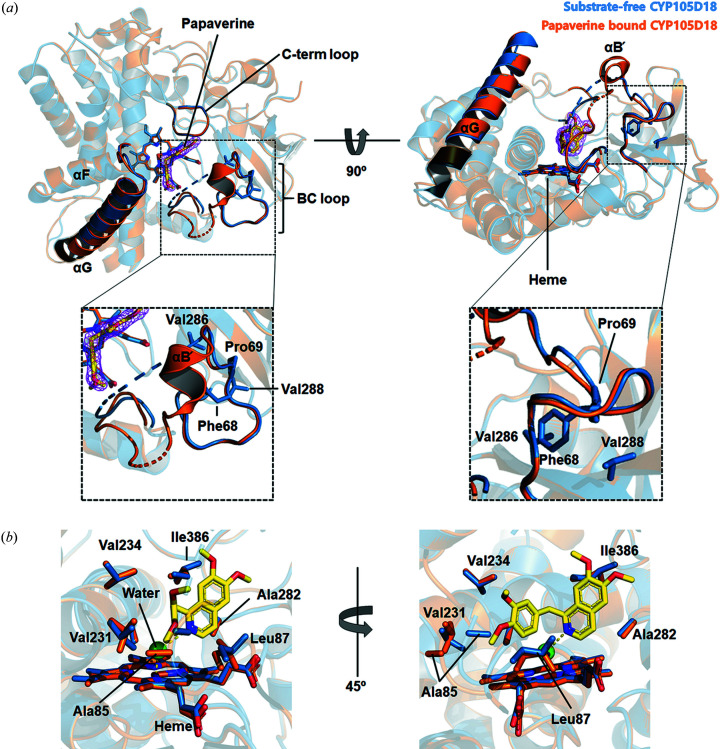
Structural comparison of the substrate-free structure and papaverine complex with CYP105D18. (*a*) Superposition of substrate-free CYP105D18 with papaverine-bound CYP105D18 colored blue and orange, respectively. The BC loop and turn of αF–αG of CYP105D18 are shown as ribbon diagrams. (*b*) Close-up view of the αB′ and hinge region of the active site. (*c*) Close-up view of the heme and papaverine-binding site. The papaverine and interacting residues are shown as stick models. Yellow stick models indicate papaverine. Bound heme molecules are also indicated by stick models.

In addition to the substrate-free structure of CYP105D18, we determined the papaverine complex structure (PDB entry 7dls) to support papaverine N-oxidation and understand the substrate-binding mode and structural changes that occur upon substrate binding. The papaverine complex structure was superimposed with the substrate-free structure and showed an overall Cα relative mean squared deviation of 0.196 Å. Structural comparisons between the substrate-free and complex forms of CYP105D18s revealed that conformation changes occurred mainly in the B/C loop region. A flexible, less structured loop was observed in the B/C loop, and electron density for residues 73–84 was absent from the substrate-free structure, indicating that this region had a high degree of flexibility. However, the papaverine complex structure had a well defined electron density for the B/C loop and formed a short helix named αB′, which has been previously observed in the diclofenac complex CYP105D7 structure (Xu *et al.*, 2015 ▸). Although the B/C loop is a less conserved region, Phe68 and Pro69 are conserved within the CYP105 family. Phe68 is located at the starting point of the B/C loop and is deeply embedded by interactions with Val286 and Val288 from β4, which may act as a hinge with Pro69 for B/C loop movement. Consistent with this observation, the electron density after Pro69 was blurry with a high *B*-factor (∼50 Å^2^), whereas the electron density for Phe68 and Pro69 showed a sharp and a good fit with a low *B*-factor [∼30 Å^2^; Fig. 7 ▸(*a*)]. Taken to­gether, these findings show that the B/C loop in the active site of the CYP105D18 structure was flexible and remained in an open confirmation without substrate. Once the substrate bound to CYP105D18, the B/C loop formed a helix (αB′) with a closed conformation. This region is thought to be responsible for substrate binding and specificity (Lee *et al.*, 2016 ▸; Xu *et al.*, 2009 ▸).

**Figure 8:**
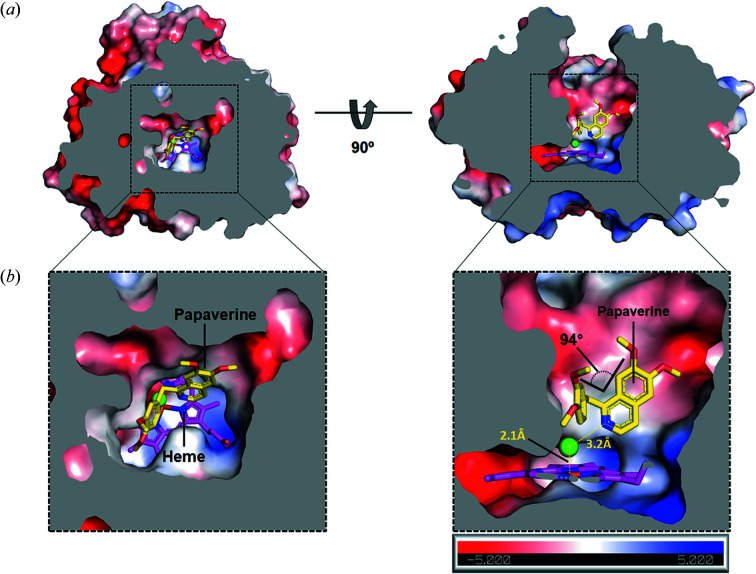
Bending motion of papaverine in the substrate-binding site of CYP105D18. (*a*) Detailed binding mode of papaverine with CYP105D18. Papaverine is at the bottom of the active site. The sphere of water molecules (green) is between the dimethodyiso­quinoline nitro­gen and iron of the heme group. Papaverine and heme are colored yellow and magenta, respectively. (*b*) Close-up view of the binding pocket in (*a*). Cross-sectional images of CYP105D18 were generated using *PyMol* (Schrödinger).

Papaverine was buried within the internal hydro­phobic cavity of CYP105D18 and was surrounded by residues Ala85, Leu87, Val231, Ala282, Val234 and Ile386, with no hydro­philic residues [Fig. 7 ▸(*b*)]. The dimethodyiso­quinoline group of papaverine was closely situated in the center of the heme, and dimethodyiso­quinoline nitro­gen was located toward the iron of the heme group. An electron-density blob corresponding to a water molecule between the iso­quinoline nitro­gen and heme iron was found (Fig. S7). The distance between the water and nitro­gen was 3.2 Å, and that between the water and iron was 2.1 Å, indicating that the binding mode represented the true mode for N-oxidation (Fig. 8 ▸). The dimethodyiso­quinoline and di­meth­oxy-phenyl groups were bent by approximately 90°, with a single carbon bond. This finding strongly supports our biochemical data demonstrating that CYP105D18 selectively transformed papaverine, although the overall biochemical characteristics of the substrates tested were similar because other iso­quinoline alkaloids are linked using two single carbon bonds that cannot be bent.

**Figure 9:**
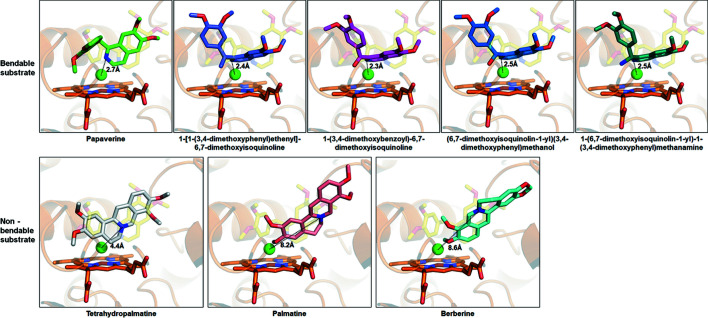
Comparison of bendable and non-bendable substrates. Co-crystallized papaverine and heme molecules with CYP105D18 are shown as yellow and orange sticks. Each potential substrate after the docking calculation was represented with sticks with different color codes. The distances between the water molecules interacting with heme in CYP105D18 and nitro­gen are indicated in each figure. Docking experiments were performed using the Lamarckian genetic algorithm (LGA) method in *Autodock Vina* (Trott & Olson, 2010 ▸).

To further support this observation, a molecular docking simulation was conducted with bendable substrates and non-bendable substrates. First, we used the papaverine model to check the reliability of the docking simulation. The simulated papaverine binds to the substrate-binding pocket of CYP105D18 with a similar binding mode to that of the co-crystallized structure (Fig. 9 ▸). The simulation results show that nitro­gen of bendable chemicals has a closer distance (2.5–2.7 Å) with water molecules interacting with iron of the heme molecule in CYP105D18 compared with those of non-bendable chemical compounds (4.4–8.6 Å). Taken together, we suggest that the bending motion of the ligand may be the critical feature in establishing the distance and coordinates during hydroxyl group transfer. Based on this analysis, we listed potential substrates for CYP105D18 in Fig. S8. Overall, this structural analysis supported the unique features of CYP105D18, including high flexibility at the active site entrance and substrate selectivity.

## 3. Conclusions   

In this study, we reported a novel cytochrome P450 enzyme annotated as CYP105D18. We observed H_2_O_2_-mediated N-oxidation of papaverine by this CYP and its high tolerance to H_2_O_2_ for papaverine biotransformation. In addition, we investigated the crystal structure of CYP105D18, which suggested that high structural similarity is shared by the CYP105 family. Structural comparison of substrate-free CYP105D18 with a substrate-bound structure revealed high flexibility at the active site entrance comprising the B/C loop. In a papaverine-bound structure, papaverine adopted a bent structure that enabled correct alignment with the heme molecule for the N-oxidation reaction of papaverine. This finding explains why CYP105D18 had strong papaverine activity but weak or no activity for other iso­quinoline alkaloids with non-bendable structures in our data. Taken to­gether, our findings showed that this novel CYP exhibited high tolerance for H_2_O_2_ and unique substrate specificity, warranting studies of substantial drug modifications for biotechnological applications.

## 4. Related literature   

The following references are cited in the supporting information: Bhattarai *et al.* (2013 ▸); DeLano (2002 ▸); Kumar *et al.* (2018 ▸); Omura & Sato (1964 ▸); Purdy *et al.* (2004 ▸); Roy *et al.* (2010 ▸); Winn *et al.* (2011 ▸); Zhang (2008 ▸).

## Supplementary Material

Experimental procedures, tables and figures. DOI: 10.1107/S2052252521005522/jt5056sup1.pdf

PDB reference: CYP105D18 complex with papaverine, 7dls

PDB reference: apo-structure of CYP105D18, 7di3

## Figures and Tables

**Table 1:** Rates of heme oxidation (*k*) for CYP105D18 in the presence of different H_2_O_2_ concentrations and comparisons with available literature ND: not detected. NP: not performed.

H_2_O_2_ conc. (m*M*)	CYP105D18 (*k* value, min^−1^)	CYP154C8 (Dangi *et al.*, 2018 ▸) (*k* value, min^−1^)	P450116B5hd (Ciaramella *et al.*, 2020 ▸) (*k* value, s^−1^)
1	ND	NP	ND
2	ND	NP	0.0024
3	ND	0.075	0.0034
4	ND	NP	0.0042
5	0.001	0.020	0.0051
10	0.003	NP	NP
20	0.004	0.120	NP
50	0.014	0.180	NP
75	0.090	0.510	NP
100	0.110	0.180	NP
200	0.230	NP	NP

**Table 2:** Structural homolog search results for CYP105D18 from a *DALI* search (*DALI-Lite* server)

CYP annotation/protein	PDB entry	*DALI* *Z*-score	UniProtKB code	Sequence % entry with CYP105D18 (aligned residue number)	Reference
CYP105D7	4ubs	53.7	Q825I8	69 (377/393)	(Xu *et al.*, 2015 ▸)
CYP105D6/PteD	3abb	53.0	Q93H80	57 (376/383)	(Xu *et al.*, 2010 ▸)
CYP105AB3/MoxA	2z36	52.3	Q2L6S8	50 (381/403)	(Miyanaga *et al.*, 2017 ▸)
GfsF	5y1i	50.8	E0D207	49 (375/388)	(McLean *et al.*, 2015 ▸)
CYP105N1/SCO7686	3tyw	49.5	Q9EWP1	43 (381/397)	Not published
CYP105A1/SuaC	2zbx	49.4	P18326	55 (375/399)	(Hayashi *et al.*, 2008 ▸)
CYP105AS1	4oqs	48.6	W5VH56	45 (366/384)	(McLean *et al.*, 2015 ▸)
CYP55A1	1f26	48.5	P23295	36 (381/399)	(Obayashi *et al.*, 2000 ▸)
CYP107E1/MycG	2ygx	47.3	Q59523	40 (381/393)	(Li *et al.*, 2012 ▸)
CYP107W1/OlmB	4wpz	47.1	Q93HJ0	39 (371/387)	(Han *et al.*, 2015 ▸)
CYP105P1/PteC	3e5j	46.1	Q93H81	44 (372/390)	(Xu *et al.*, 2009 ▸)
