# Supplementary material for: Characterization of high-H2O2-tolerant bacterial cytochrome P450 CYP105D18: insights into papaverine N-oxidation
Source: IUCrJ. 2021 Jun 29;8(Pt 4):684–94. doi: 10.1107/S2052252521005522 (PMC8256718; doi:10.1107/S2052252521005522)
Supplement: Supplementary file 1 [file m-08-00684-sup1.pdf]

IUCrJ

Volume 8 (2021)

Supporting information for article:

Characterization of high-H2O2-tolerant bacterial cytochrome P450 CYP105D18: insights into papaverine *N*-oxidation

Bashu Dev Pardhe, Hackwon Do, Chang-Sook Jeong, Ki-Hwa Kim, Jun Hyuck Lee and Tae-Jin Oh

Table of Contents

Supplemental Experimental Procedures
Supplemental Table S1. HPLC conditions for papaverine.
Supplemental Table S2. HPLC conditions for tetrahydropalmatine, berberine, and palmatine.
Supplemental Table S3. X-ray diffraction data collection and refinement statistics.
Supplemental Figure S1. Phylogenetic analysis of the CYP105 family with CYP105D18.
Supplemental Figure S2. SDS-PAGE analysis for Pdx and PdR.
Supplemental Figure S3. Isoquinoline alkaloids used for *in vitro* reaction screening.
Supplemental Figure S4. Structural elucidation of papaverine *N*-oxide by NMR analysis.
Supplemental Figure S5. Papaverine-bound spectral analysis for CYP105D18.
Supplemental Figure S6. Molecular mass of CYP105D18 determined based on a standard curve obtained using a Superdex 10/300 GL column.
Supplemental Figure S7. Stereoview of papaverine binding site.
Supplemental Figure S8. Potential CYP105D18 substrates.

Experimental Procedures

Chemicals and reagents
Substrates, papaverine hydrochloride, tetrahydropalmatine, berberine, and palmatine (>99% high-performance liquid chromatography [HPLC] grade) were purchased from Tokyo Chemical Industry Co., Ltd. (Tokyo, Japan). T4 DNA ligase, DNA polymerase, and dNTPs were obtained from Takara Bio (Shiga, Japan). 5α-Aminolevulinic acid hydrochloride, hydrogen peroxide (H_2O_2), (diacetoxyiodo)benzene, ampicillin, nicotinamide adenine dinucleotide (NADH), nicotinamide adenine dinucleotide phosphate (NADPH), catalase, formate dehydrogenase, sodium formate, spinach ferredoxin (Fdx), and spinach ferredoxin reductase (FdR) were obtained from Sigma-Aldrich (St. Louis, MO, USA). Isopropyl-1-thio-β-D-galactopyranoside (IPTG) and kanamycin were purchased from Duchefa Bohemia (Haarlem, Netherlands). Restriction enzymes were procured from Takara Clontech.

Genomic analysis
The complete genome sequence of thiostrepton-producing *Streptomyces laurentii*, strain ATCC31255 (GenBank: AP017424.1), comprised 29 gene-coding putative CYPs. The gene encoding CYP105D18 was annotated as *SLA_5925*. The phylogenetic tree of CYP105 family homologs in *Streptomyces* was constructed using MEGA X software with the maximum likelihood tree (Kumar *et al*., 2018). The percentages of replicate trees in which the associated taxa clustered together in the bootstrap test (500 replicates) were evaluated. The tree is drawn to scale, with branch lengths in the same units as the evolutionary distances used to infer the phylogenetic tree. The analysis involved all CYP105 superfamily proteins included by Dr. Nelson (http://drnelson.uthsc.edu/Cytochrome P450.html) and other 105D subfamilies that have been given a specific nomenclature by Dr. Nelson upon our request from the bacterial strain *S. laurentii*.

Cloning and overexpression of CYPs and redox partners
CYP105D18, encoding 396 amino acids (GenBank; BAU86794.1) from the strain *S. laurentii*, was amplified using the specific PCR forward primer 5′-GAATTCATGACCGAAGCCGTGG-3′ (EcoRI) and reverse primer 5′-AAGCTTTCCGCCTACCAGGTGA-3′ (HindIII). The purified PCR product was cloned into the pMD20-T vector using *Escherichia coli* XL1-Blue and selected with blue-white screening, and the nucleotide sequences were confirmed by automated sequencing (Macrogen, Seoul, Korea). *CYP105D18* was ligated into pET28a(+), and the construct was transformed into *E. coli* XL1-Blue. The construct pET28aCYP105D18 was plated on LB agar containing 100 μg/mL kanamycin. The amplified construct DNA encoding N-terminal His6-tag protein under the control of a T7 promoter was isolated and transformed into chemically competent C41 cells for overexpression. The cells were plated on LB agar containing 100 μg/mL antibiotic. Seed cultures (3 mL) were grown for 3 h from a single colony under stress (100 μg/mL antibiotic), and 0.3 mL seed culture was added to 100 mL LB medium supplemented with 100 μg/mL antibiotics and incubated on an orbital shaker (180 rpm) at 37°C until the cell density was approximately 0.6 at OD_{600}. The culture was supplemented with 1 mM 5α-aminolevulinic acid hydrochloride and 0.5 mM $FeCl_3$ to support heme synthesis and grown for an additional 1 min. Induction was performed with 0.4 mM IPTG, followed by incubation for 72 h at 20 °C for protein synthesis. The cell pellets were harvested by centrifugation (3,500 rpm) for 30 min at 4 °C, washed twice with 50 mM potassium phosphate buffer (pH 7.4), and stored at -50 °C. Different redox partners supported the *in vitro* reconstituted system. Redox partners putidaredoxin reductase (camA) and putidaredoxin (camB) were overexpressed as His383-tagged proteins in *E. coli* BL21(DE3) using plasmid constructs pET28a(+) and pET32a(+), as described previously (Bhattarai *et al*., 2013). Cells were harvested by centrifugation (3,500 rpm) for 30 min at 4 °C, washed twice with 50 mM potassium phosphate buffer (pH 7.4), and stored at −50 °C.

Purification of CYP105D18 and redox partners

Harvested cell pellets were suspended in potassium phosphate buffer (pH 7.4) and lysed by ultrasonication. The soluble protein-containing fraction was separated by centrifugation at 24,650 ×*g* for 20 min at 40 °C and purified by Ni^{2+} affinity chromatography using a TALON His-tag. Resin-bound proteins were eluted using elution potassium buffer (7.4) containing 10% glycerol, 100 mM NaCl, and different concentration gradients of imidazole (10, 100, and 250 mM). The purity of the protein was checked by sodium dodecyl sulfate-polyacrylamide gel electrophoresis. All fractions and purified fractions were concentrated by ultrafiltration with Amicon centrifugal filters with a molecular weight cut-off of 30 kDa for CYP105D18 and putidaredoxin reductase (PdR) and 10 kDa for putidaredoxin (Pdx).

Analytical size-exclusion chromatography

To estimate the oligomeric state of CYP105D18, size-exclusion chromatography was carried out at 22 °C using an AKTA FPLC system and Superdex 200 10/300 GL column from GE Healthcare (Little Chalfont, UK). The column was equilibrated in 50 mM phosphate buffer (pH 8), 150 mM NaCl, and 1 mM DTT at a flow rate of 0.4 mL min^{-1}.

Determination of enzyme concentrations

The CYP105D18 concentration was determined by the CO difference spectra, as described previously. The amount of CYP was calculated from $\varepsilon_{449–489}$ = 91 mM^{-1} cm^{-1} (Omura and Sato, 1964). The PdR concentration was determined as the average concentration calculated from wavelengths 378, 454, and 480 nm using extinction coefficients (ε) of 9.7, 10.0, and 8.5 mM^{-1} cm^{-1}, respectively, and the Pdx concentration was also determined as the average of concentrations calculated from wavelengths 415 and 454 nm using extinction coefficients of 11.1 and 10.4 mM^{-1} cm^{-1}, respectively (Purdy *et al.*, 2004). To determine purity (RZ value), the sample containing CYP was scanned in the range of 200–500 nm; the reference contained only buffer. All samples were scanned with a Biochrom Libra S35PC UV/visible spectrophotometer (Cambridge, UK).

In vitro *biotransformation by CYP105D18*

The *in vitro* reactions for CYP105D18 were assisted with redox partners (PdR-Pdx and FdR-Fdx systems) and oxygen surrogates H_2O_2 and diacetoxyidodo(benzene). All *in vitro* biotransformations were carried out in a 400-µL reaction mixture for papaverine, tetrahydropalmatine, palmatine, and berberine with 3 µM CYP in 50 mM phosphate buffer (pH 7.4). Berberine, tetrahydropalmatine, and palmatine were prepared in 100 mM stock in dimethyl sulfoxide (DMSO). Papaverine was dissolved in ethyl alcohol and used at a concentration of less than 1% (v/v) in the reaction mixture. The reaction mixture contained CYP, PdR, and Pdx at a ratio of 1:2:16, 100 mg/mL catalase, 1 mM $MgCl_2$, 400 µM substrate, and NADH regeneration system (1 U formate dehydrogenase and 150 mM sodium formate). Similarly, CYP, FdR, and Fdx were used at a ratio of 1:2:10 with an NADPH regeneration system (glucose dehydrogenase and glucose). The reaction was initiated by NAD(P)H and incubated for 1 h at 30 °C with vigorous shaking at 500 rpm. The reaction was optimized by adding different concentrations of oxygen surrogates for stochastic use to achieve maximum catalytic efficiency. Reactions were initiated by 1–800 mM H_2O_2 and 0.5–5 mM diacetoxyidodo(benzene).

H_2O_2 tolerance of CYP105D18

The heme oxidation rate was determined using H_2O_2 concentrations ranging from 1 to 200 mM. CYP (3 µM) was prepared in 1 mL of 50 mM potassium phosphate buffer. After adding H_2O_2, the absorbance was recorded at a wavelength of 350–500 nm every 90 s for 30 min. The Soret peak absorbance at 417 nm was plotted against time, and the heme oxidation rate constant (k) was calculated using one-phase decay in GraphPad Prism 5 software. Associated absorbance amplitudes (A) were calculated as the differences between the highest and lowest absorbances at 417 nm. All samples were scanned using a Biochrom Libra S35PC UV/visible spectrophotometer.

Product extraction, purification, and analysis

The reactions were extracted using an equal volume of ethyl acetate twice, dried, and dissolved in HPLC-grade methanol for further analysis. The mixture was filtered with a 0.2-µm Whatman filter and injected into an ultra-HPLC instrument and separated using a Mightysil reverse-phase C18 column (4.6 × 250, 5 µm; Kanto Chemical, Tokyo, Japan). The gradient system and solvents used to separate the different compounds are described in Supplemental Tables S1 and S2. Following HPLC analysis, the reaction mixtures were analyzed by SYNAPT G2-S/ACUITY UPLC liquid-chromatography quadrupole time-of-flight/electrospray ionization mass spectrometry (Waters, Milford, MA, USA) in positive ion mode. A large-scale reaction (100 mL), with each 15 mL containing 3 µM CYP, 500 µM papaverine, and 40 mM H_2O_2, was carried out for 1 h and extracted with a double volume of ethyl acetate for structural analysis by nuclear magnetic resonance (NMR). The purified product was dissolved in DMSO and subjected to NMR analysis (800 MHz) with a Varian Unity INOVA spectrometer (Varian, Palo Alto, CA, USA). One-dimensional NMR (^{1}H NMR and ^{13}C NMR) was performed, followed by 2D NMR, HMBC, ROESY, and HSQC to elucidate the exact structures when appropriate.

Determination of kinetic parameters

The time-dependent *in vitro* conversion of papaverine to papaverine N-oxide was measured every 5 min for 1 h using 1 µM CYP105D18, 40 mM H_2O_2, and 400 µM substrate. All reaction mixtures were extracted as described previously. The conversion (%) of each product at different time intervals was calculated from the area of the product peaks based on the HPLC chromatogram. The product concentration with the substrate papaverine in the presence of 40 mM H_2O_2 was determined at different substrate concentrations of 5–500 µM. The reaction mixture was incubated and extracted as described elsewhere. Assuming that the absorbance properties of products and substrates were the same, the products were quantified by correlating the respective product peak areas with the combined peak areas of the products and substrate. K_m and k_{cat} values were calculated by plotting the product

formation rate against the substrate concentration. Experiments were carried out three times under the same optimized conditions, and data were analyzed to calculate the average conversion rate and kinetic parameters.

Crystallization and data collection

Crystallization was performed with 52.7 mg/mL recombinant CYP105D18 using the hanging drop vapor diffusion method. The CYP105D18 crystals grew under conditions of 0.2 M lithium sulfate, 0.1 M Tris-HCl, pH 8.5, and 25% (w/v) PEG 3350 at room temperature after 2 days and were further optimized to obtain diffraction-quality crystals. For papaverine complex crystals, the protein was mixed with papaverine at a 1:5 ratio and initially screened. The papaverine-CYP complex crystals were obtained under the following conditions: 0.1 M Tris-HCl, pH 8.5, 1.5 M ammonium phosphate dibasic. After incubating the crystals in paratone as a cryoprotectant for 10 min, they were frozen in liquid nitrogen. Native diffraction data were collected using a BL-5C beamline 5A instrument at PLS (Korea).

Structure determination and refinement

Data processing and reduction were performed using HKL2000, and the structure phase from the CYP105D7 structure (PDB: 4UBS) was obtained by molecular replacement using the program MOLREP from the CCP4i suite (Winn *et al.*, 2011). The coordinate of CYP105D18 was built by iterative model building using Coot and refinement using Refmac5 and Phenix.refine program. The quality of the final model was verified using Molprobity, and a Molprobity score in the 100th percentile was obtained (Williams *et al.*, 2018). All structure figures were prepared using PyMOL (DeLano, 2002).

Docking simulation

The papaverine-complex structure without crystallographic water molecules and papaverine were used templates for the docking experiment. Model structures of substrate were built with JLigand (Lebedev *et al*., 2012). Atomic charge and rotatable bonds are set using the method described in the Autodock tools package (Trott *et al*., 2010). Docking experiments were performed using the Lamarckian Genetic Algorithm (LGA) method implemented in Autodock 4. The best model from the experiments was used as a representative model.

Supplemental Tables and Figures

Supplemental Table S1. HPLC conditions for papaverine. Solvent A, 0.05% acetic acid water; solvent B, acetonitrile

Time (min)	Flow (mL min^{-1})	%B
**Equilibration**		
**0.0**	1.00	0.0
**Run**		
**5**	1.00	10
**15**	1.00	40
**20**	1.00	90
**22**	1.00	90
**25**	1.00	10
**30**	Stop	

Supplemental Table S2. HPLC conditions for tetrahydropalmatine, berberine, and palmatine. Solvent A, 10 mM ammonium acetate, and 0.2% triethylamine in water, pH 5.0, set with acetic acid; and solvent B, acetonitrile

Time (min)	Flow (mL min^{-1})	%B
**Equilibration**		
**0.0**	1.00	0.0
**Run**		
**5**	1.00	10
**15**	1.00	40
**20**	1.00	90
**22**	1.00	90
**25**	1.00	10
**30**	Stop	

Supplemental Table S3. X-ray diffraction data collection and refinement statistics.

Dataset	Apo-CYP105D18	Papaverine complex
X-ray source	BL-5C beamline	BLa-5C beamline
Space group	$C2_1$	$C2_1$
Unit-cell parameters (Å, °)	a = 91.68, b = 52.44, c = 87.95, α = γ = 90.00, β = 110.82	a = 91.52, b = 52.17, c = 87.89, α = γ = 90.00, β = 110.68
Wavelength (Å)	0.9794	0.9794
Resolution (Å)	50.00–1.70 (1.73–1.70)	50.00–2.06 (2.10–2.06)
Unique reflections	78248 (3385)	41490 (1697)
Average I/σ (I)	29.4 (3.5)	17.05 (2.96)
R_{merge} [a]	0.074 (0.360)	0.045 (0.233)
Redundancy	4.9 (3.2)	4.7 (2.8)
Completeness (%)	96.9 (92.6)	94.15 (84.70)
Refinement		
Resolution range (Å)	27.42–1.69 (1.73–1.69)	44.59–2.06 (2.11–2.06)
No. of reflections of working set	40181 (2470)	21580 (1433)
No. of reflections of test set	2211 (138)	1127 (81)
No. of amino acid residues	381	392
No. of water molecules	329	144
Molecules per asymmetric unit	1	1
R_{cryst} [b]	0.16 (0.23)	0.18 (0.25)
R_{free} [c]	0.21 (0.24)	0.21 (0.29)
R.m.s. bond length (Å)	0.011	0.015
R.m.s. bond angle (°)	1.750	1.86
Average B value ($Å^2$) (protein)	27.49	35.09
Average B value ($Å^2$) (solvent)	34.98	37.27

[a] $R_{merge} = \sum |\langle I\rangle - I| / \sum \langle I\rangle$.

[b] $R_{cryst} = \sum ||Fo| - |Fc|| / \sum |Fo|$.

[c] R_{free} calculated with 5% of all reflections excluded from refinement stages using high-resolution data.

Values in parentheses refer to the highest resolution shells.

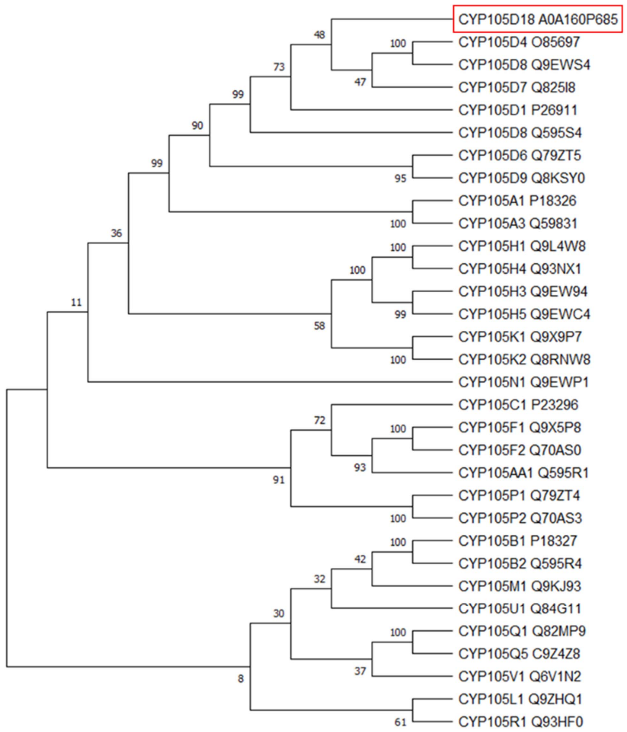

Supplemental Figure S1. Phylogenetic analysis of CYP105 family members with the CYP105D18 Unicode for each protein. The phylogenetic tree was obtained in Mega-X using the neighbor-joining method (Kumar *et al.*, 2018). The values above the branches indicate confidence percentages based on a bootstrap value of 500 repetitions.

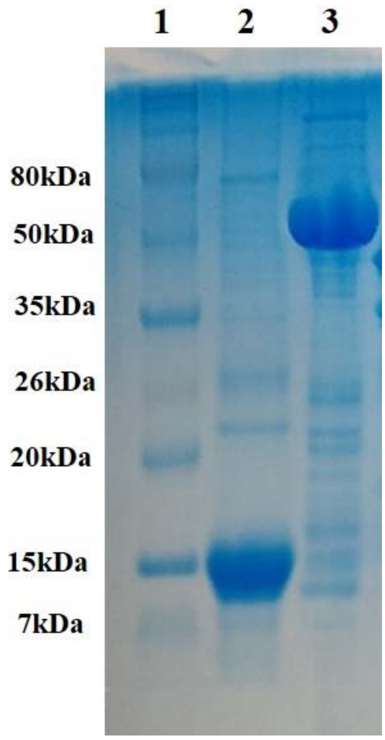

Supplemental Figure S2. SDS-PAGE analysis of Pdx and PdR. The theoretical molecular weight of Pdx is 11.4 kDa, and the protein was overexpressed with the pET28(+) vector in a C41 host. The theoretical molecular weight of PdR is 45.6 kDa, and the protein was overexpressed with the pET32a(+) vector in a C41 host. 1, protein marker; 2, soluble fraction of Pdx; and 3, soluble fraction of PdR.

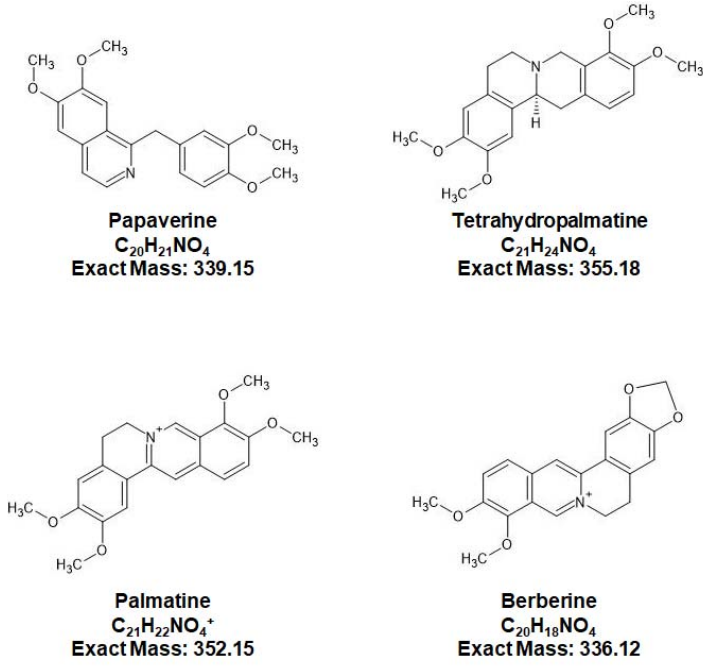

Supplemental Figure S3. Isoquinoline alkaloids used for *in vitro* reaction screening.

^{1}H NMR of papaverine *N*-oxide

^{1}H NMR (700 MHz, DMSO) δ 8.13 (d, *J* = 7.1 Hz, 1H), 7.66 (d, *J* = 7.1 Hz, 1H), 7.40 (s, 1H), 7.38 (s, 1H),7.16 (d, *J* = 2.0 Hz, 1H), 6.84 (dd, *J* = 8.2, 2.0 Hz, 1H), 6.80 (d, *J* = 8.2 Hz, 1H), 4.67 (s, 2H), 3.91 (s, 3H), 3.89 (s, 3H), 3.68 (s, 3H), 3.67 (s, 3H).

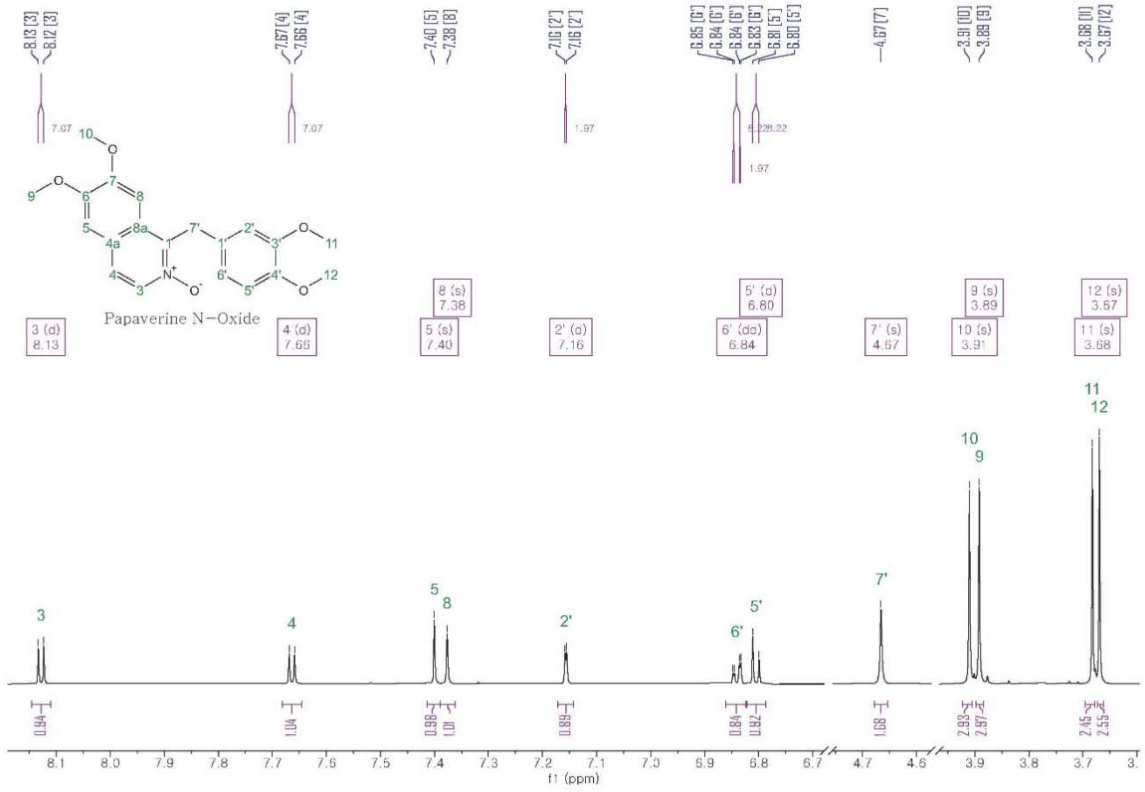

^{13}C NMR of papaverine *N*-oxide

^{13}C NMR (176 MHz, DMSO) δ 151.72, 150.88, 149.07, 147.82, 145.26, 135.26, 131.19,124.98, 124.58, 121.72, 120.84, 113.27, 112.44, 107.09, 103.50, 56.33, 56.25, 55.94, 55.87,30.73.

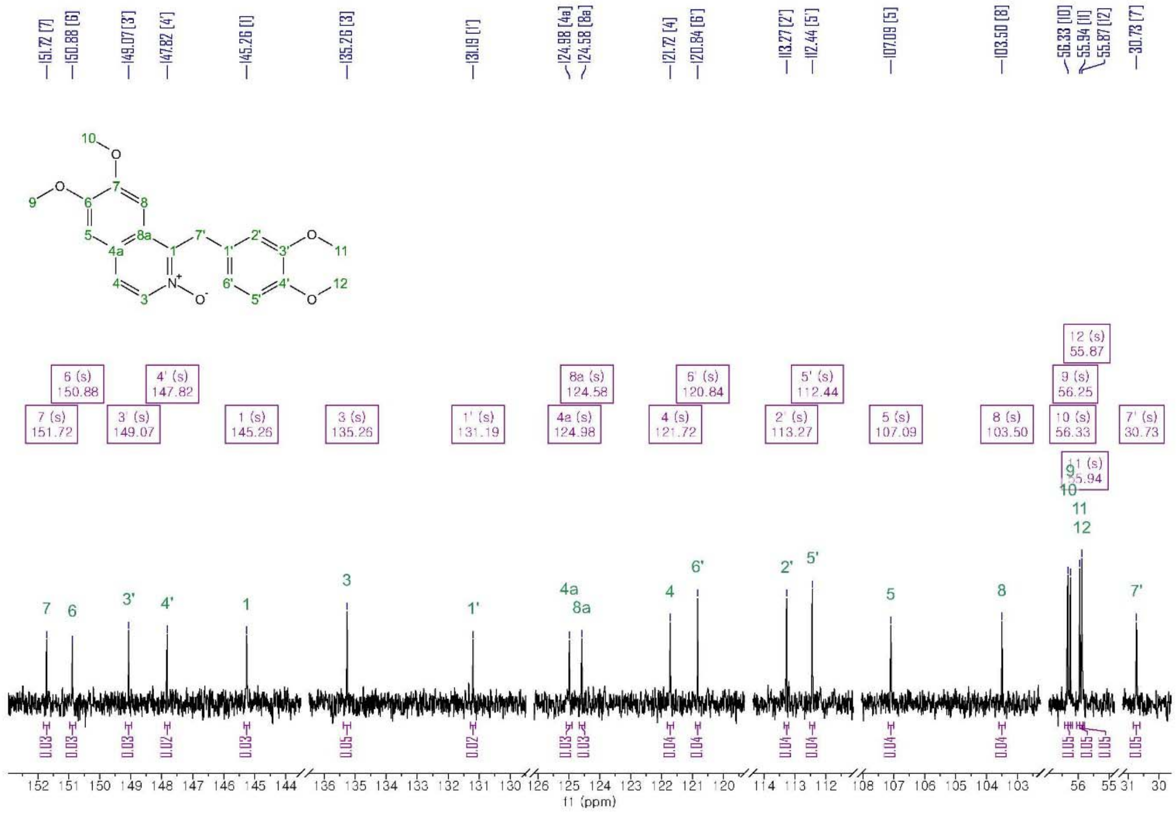

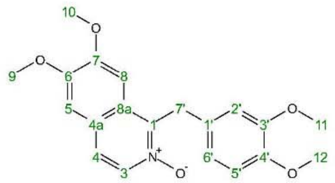

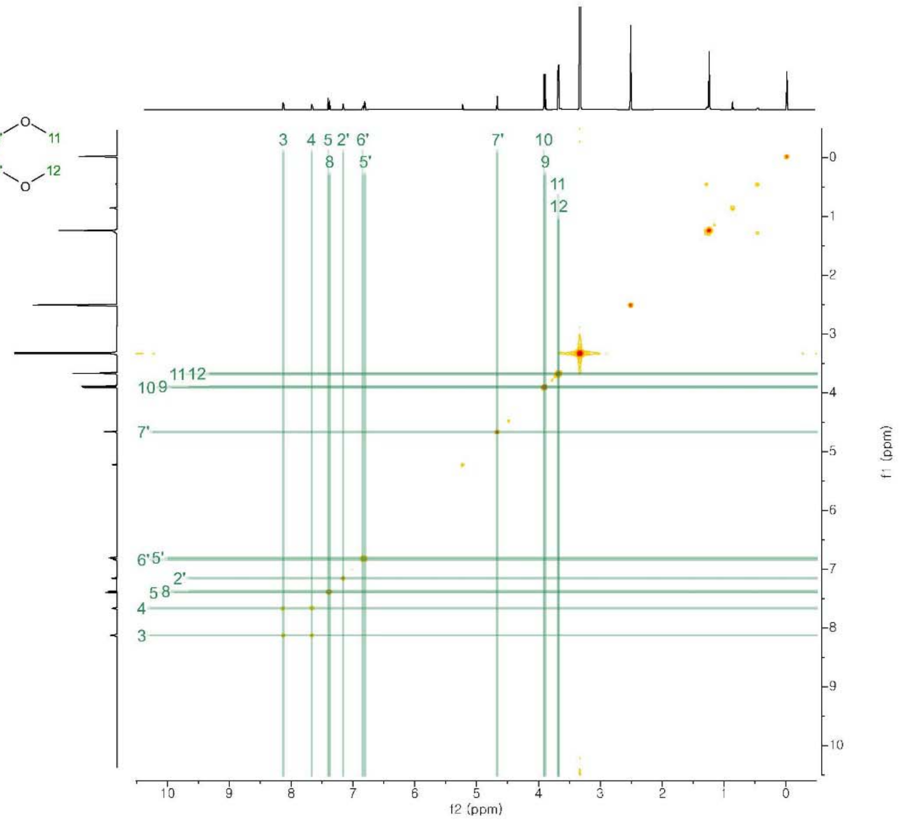
f2 (ppm)
f1 (ppm)

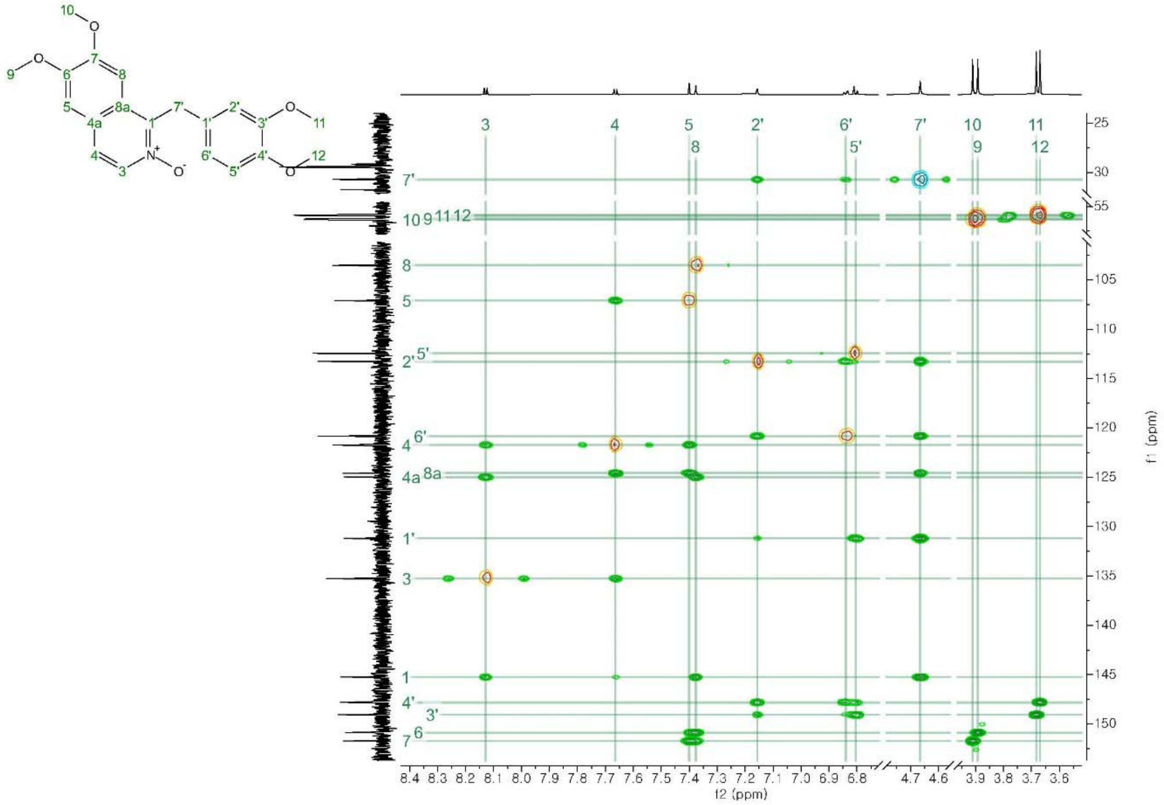

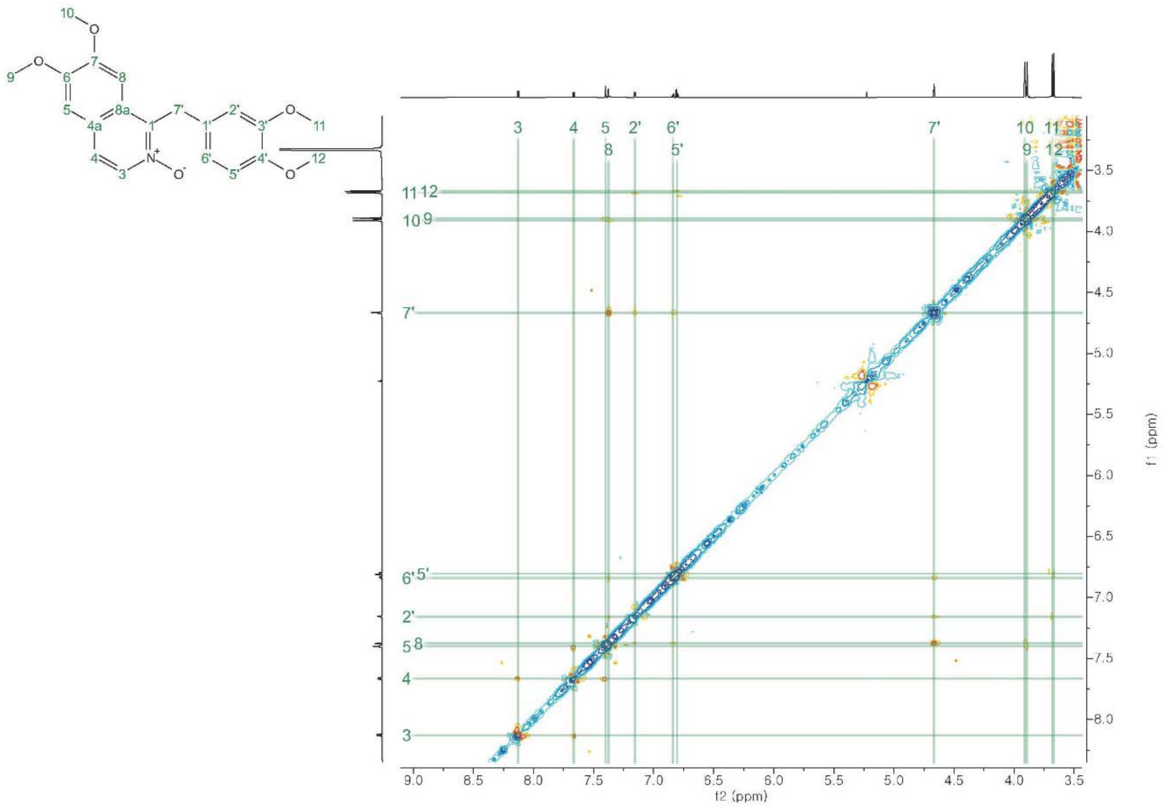

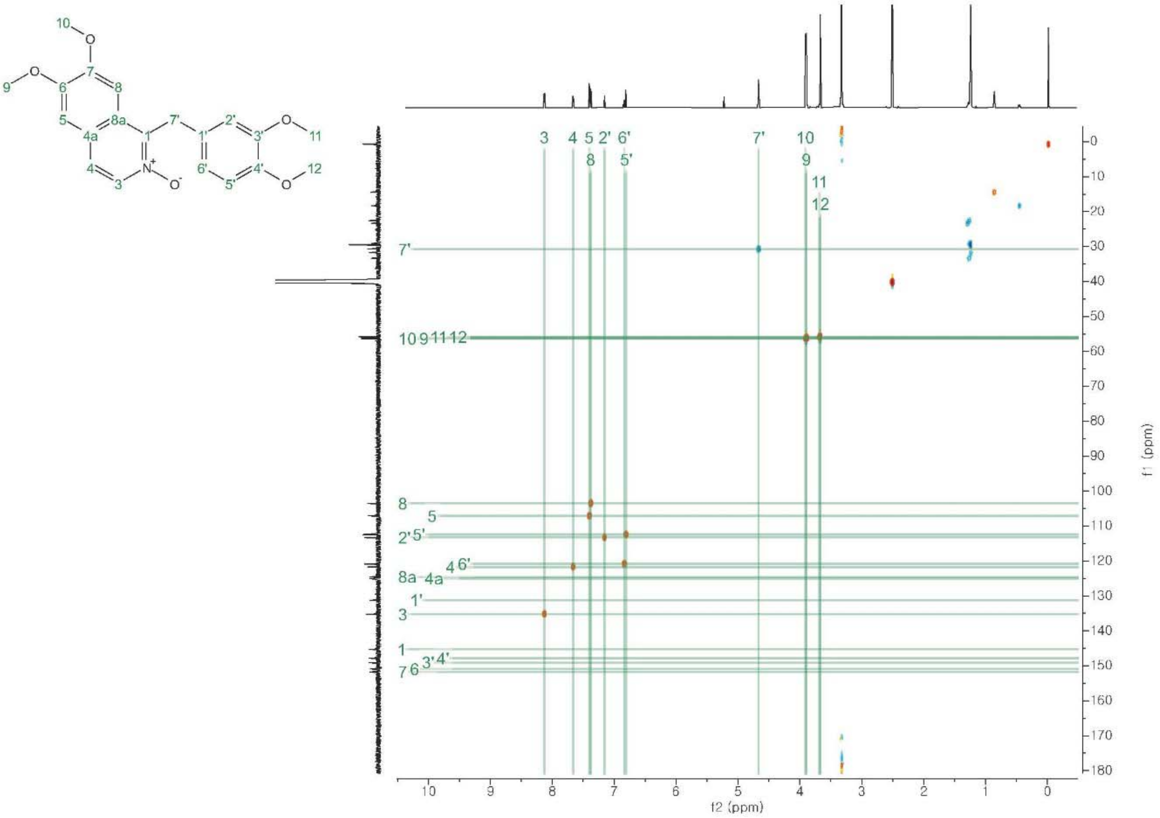

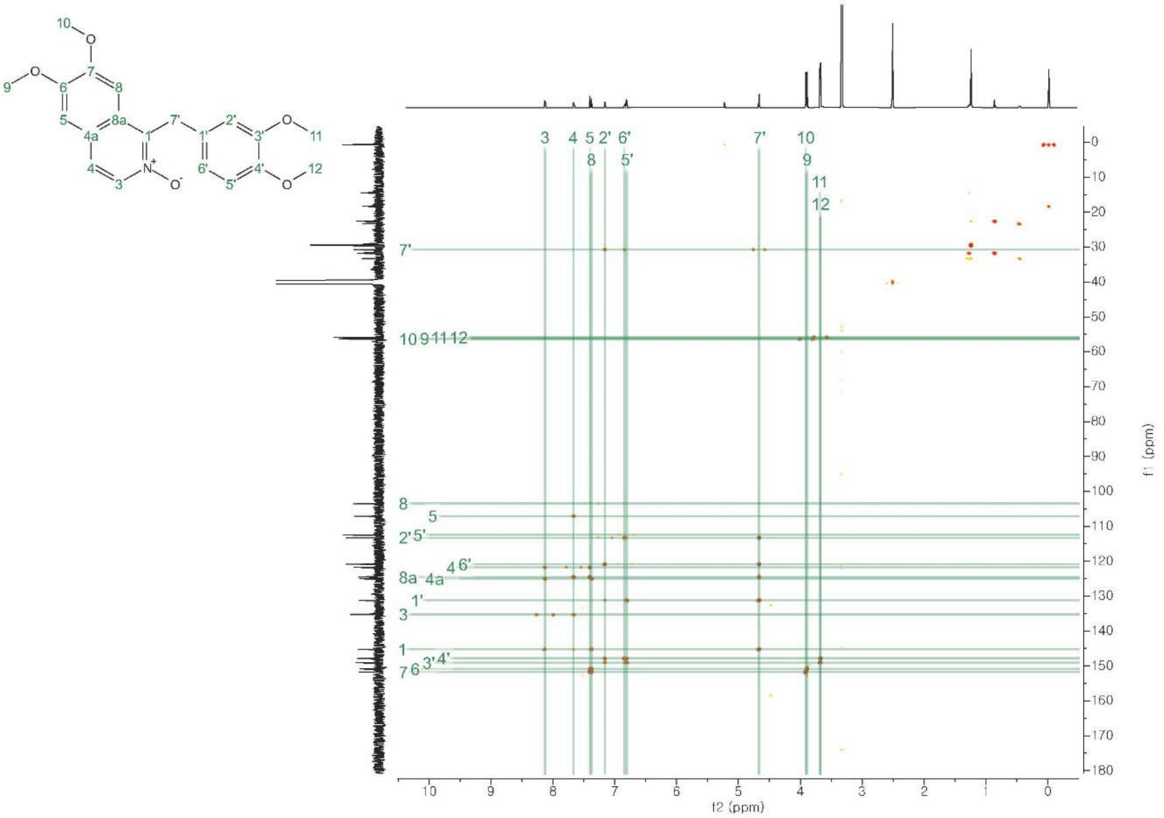

Supplemental Figure S4. Structural elucidation of papaverine *N*-oxide.

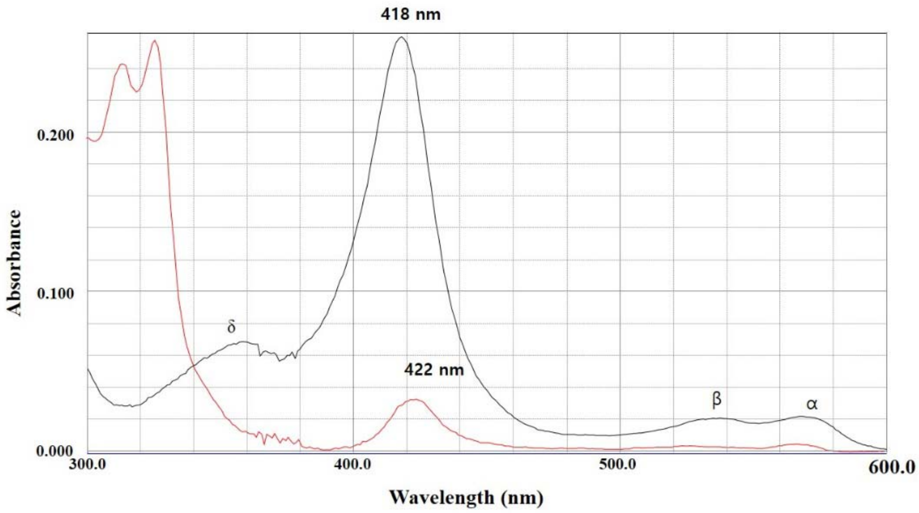

Supplemental Figure S5. Papaverine-bound spectral analysis for CYP105D18. CYP105D18 induced Soret wavelength at 418 nm (black), and CYP105D18, papaverine-bound spectral shift to 422 nm (red). The concentration of CYP105D18 used was 3μm.

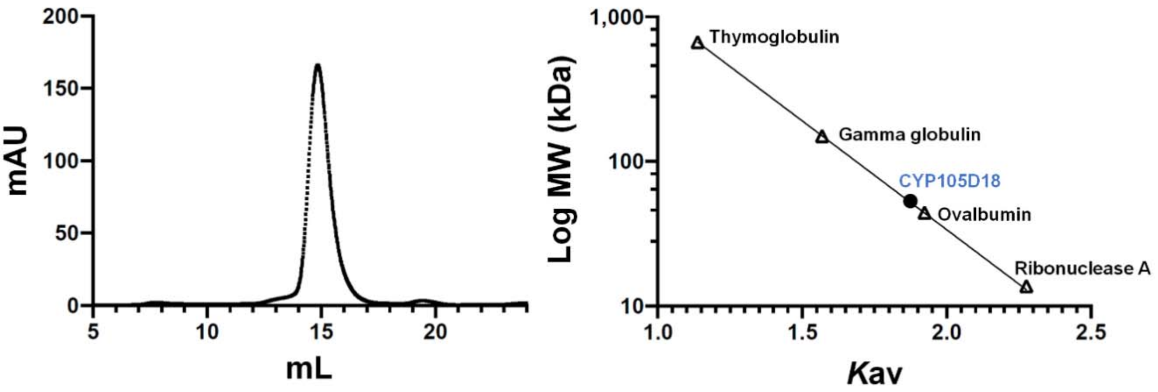

Supplemental Figure S6. The molecular mass of CYP105D18 was determined based on a standard curve obtained using a Superdex 200 10/300 GL column. The size of CYP105D18 was estimated to be 53 kDa. The molecular weight standard for gel filtration chromatography was calibrated with thymoglobulin (670 kDa), γ-globulin (158 kDa), ovalbumin (44 kDa), and ribonuclease A (17 kDa). The elution volume was used to calculate the partition coefficient, *K*av = (Ve / Vo) / (Vt / Vo).

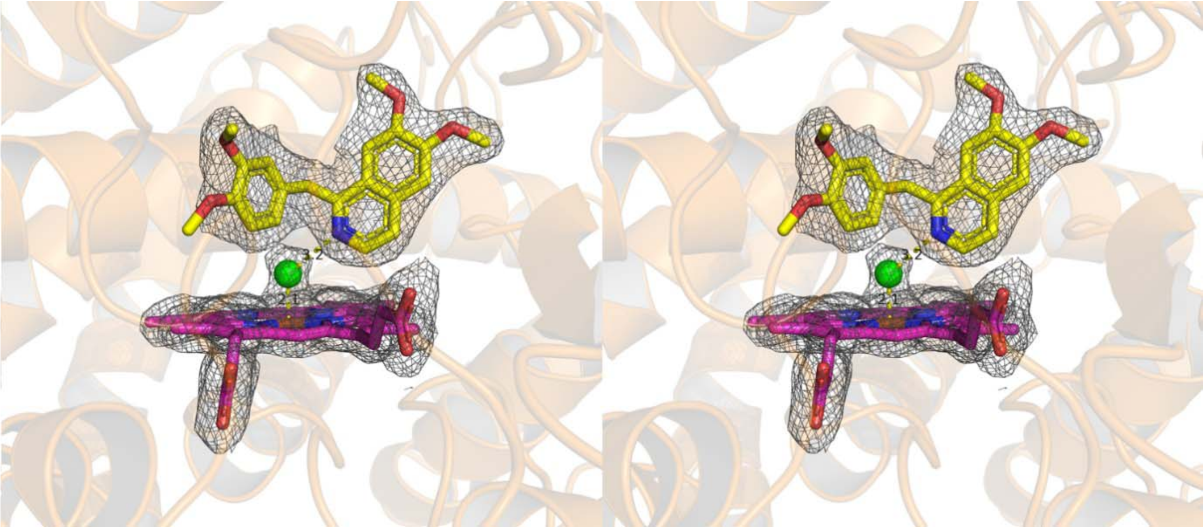

Supplemental Figure S7. Stereo-view of papaverine binding site. Papaverine and heme are shown in yellow and magenta, respectively. Water molecules are shown in green. The 2Fo-Fc omit maps for papaverine, heme, and water contoured at 2 σ are shown as black lines. Hydrogen bonds are depicted as yellow dots with distances.

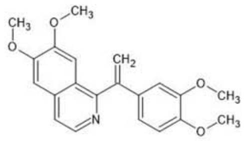

1-[1-(3,4-dimethoxyphenyl)ethenyl]-6,7-dimethoxyisoquinoline

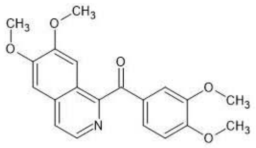

1-(3,4-dimethoxybenzoyl)-6,7-dimethoxyisoquinoline

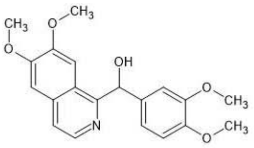

(6,7-dimethoxyisoquinolin-1-yl)(3,4-dimethoxyphenyl)methanol

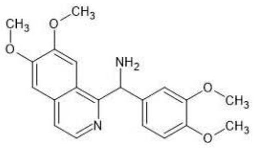

1-(6,7-dimethoxyisoquinolin-1-yl)-1-(3,4-dimethoxyphenyl)methanamine

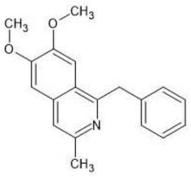

1-benzyl-6,7-dimethoxy-3-methylisoquinoline

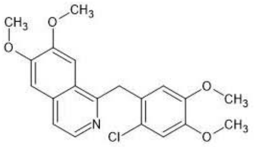

1-[(2-chloro-4,5-dimethoxyphenyl)methyl]-6,7-dimethoxyisoquinoline

Supplemental Figure S8. Potential CYP105D18 substrates.

References

Bhattarai, S., Liou, K. & Oh, T. J. (2013). *Arch. Biochem. Biophys.* **539**, 63–69.

DeLano, W. L. (2002). *Protein Crystallogr.* **40**, 1–8.

Kumar, S., Stecher, G., Li, M., Knyaz, C. & Tamura, K. (2018). *Mol. Biol. Evol.* **35**, 1547–1549.

Omura, T. & Sato, R. (1964). *J. Biol. Chem.* **239**, 2370–2378.

Purdy, M. M., Koo, L. S., Ortiz De Montellano, P. R. & Klinman, J. P. (2004). *Biochemistry.* **43**, 271–281.

Roy, A., Kucukural, A. & Zhang, Y. (2010). *Nat. Protoc.* **5**, 725–738.

Trott, O. & Olson, A. (2010). *J. Comp. Chem.* **31**, 455–461.

Williams, C. J., Headd, J. J., Moriarty, N. W., Prisant, M. G., Videau, L. L., Deis, L. N., Verma, V., Keedy, D. A., Hintze, B. J., Chen, V. B., Jain, S., Lewis, S. M., Arendall W. B. 3rd, Snoeyink, J., Adams, P. D., Lovell, S. C., Richardson, J. S. & Richardson, D. C. (2018). *Protein Sci.* **27**, 293–315.

Winn, M. D., Ballard, C. C., Cowtan, K. D., Dodson, E. J., Emsley, P., Evans, P. R., Keegan, R. M., Krissinel, E. B., Leslie, A. G. W., McCoy, A., McNicholas, S. J., Murshudov, G. N., Pannu, N. S., Potterton, E. A., Powell, H. R., Read, R. J., Vagin, A. & Wilson, K. S. (2011). *Acta. Crystallogr. D Biol. Crystallogr.* **67**, 235–242.

Zhang, Y. (2008). *BMC Bioinformatics*. **9**, 40.
